# Bus Travel Time Prediction Model Based on Profile Similarity

**DOI:** 10.3390/s19132869

**Published:** 2019-06-28

**Authors:** Teresa Cristóbal, Gabino Padrón, Alexis Quesada-Arencibia, Francisco Alayón, Gabriel de Blasio, Carmelo R. García

**Affiliations:** Institute for Cybernetics, University of Las Palmas de Gran Canaria, Campus de Tafira, 35017 Las Palmas, Spain

**Keywords:** road-based mass transit systems, intelligent transport systems, travel time prediction, clustering, automatic vehicle location

## Abstract

In road-based mass transit systems, travel time is a key factor in providing quality of service. This article proposes a method of predicting travel time for this type of transport system. This method estimates travel time by taking into account its historical behaviour, represented by historical profiles, and the current behaviour recorded on the public transport vehicle for which the prediction is to be made. The model uses the *k*-medoids clustering algorithm to obtain historical travel time profiles. A relevant feature of the model is that it does not require recent travel time data from other vehicles. For this reason, the proposed model may be used in intercity transport contexts in which service planning is carried out according to timetables. The proposed model has been tested with two real cases of intercity public transport routes and from the results obtained we may conclude that, in general, the average error of the predictions is around 13% compared to the observed travel time values.

## 1. Introduction

In road-based mass transit systems, travel time (*TT*) estimates are used for various key tasks. In operations’ planning, suitable time estimates are required for the preparation of timetables and to schedule services on the different routes. This type of planning is called long-term travel time planning. In operations’ monitoring, depending on the situation of the public transport vehicles and the conditions under which they operate the routes, travel time estimates are required to detect any potential significant deviations from the timetable and the scheduled services. This type of planning is called short-term *TT* planning. According to the standards and recommendations of public transport agencies [1], *TT* is one of the key factors when providing quality of service. Users wish to travel on the public transport network in the shortest possible time using punctual services, and to be informed in the event of alterations in order to avoid waiting [2].

In the context of road-based mass transit systems, short-term travel time forecasts estimate the time a public transport vehicle will take to reach points on the route that it has not yet passed. Due to a variety of different factors, such as demand, traffic conditions, weather conditions, etc., the development of models that provide reliable short-term *TT* predictions is a research challenge. In addition, for these types of predictions to be useful, they must be done in the shortest possible time, which means that the real-time parameter is an important feature. There are multiple studies that have proposed short-term *TT* prediction models. The common denominator in these studies is the fact that the proposed solutions take into account historical *TT* behaviour, modelled using different automatic learning techniques or statistics, and current *TT* behaviour on the route section for which *TT* is to be predicted. In these models, it is assumed that current *TT* behaviour resembles the *TT* observed in those vehicles that have recently travelled the section for which they want to make the prediction. This assumption about current *TT* behaviour is only possible when there is a planning of line services that guarantees that the sections for which *TT* is to be estimated will be covered by public transport vehicles with a certain frequency. In general, this situation occurs in the case of urban public transport systems where trips are planned by frequency of stops. However, in the case of intercity public transport, which is planned by timetable, it is not always possible to have representative *TT* values recently obtained from other vehicles. For this reason, most of the models proposed in the literature are applicable in contexts in which the routes of public transport vehicles are planned by frequency. But nowadays, especially with public transport service planning models that take into account last-mile problems in rural areas, it is also necessary to make *TT* predictions for intercity transport, since these models require synchronisation between buses and other types of vehicles, such as taxis.

In this paper, a short-term *TT* prediction model based on profile similarity is proposed. The proposed prediction model is based on the similarity between historical *TT* behaviour, represented by representative profiles obtained through clustering techniques, and *TT* behaviour observed in the vehicle itself. This paper contains four main contributions. The first is that the proposed method may be used for predicting *TT* in contexts where there is no recent *TT* information for other vehicles; it can therefore be used in the case of intercity transport. Second, the prediction may be made autonomously in the vehicle itself without the need to communicate with a control centre. Third, due to the computational cost and parameterisation of the techniques used, the proposed *TT* prediction model may be applied continuously to all the routes of a transport network at less cost than alternative methods, which generally require more complex learning and testing processes. Fourth, due to the way that historical *TT* behaviour is represented, the model not only provides short-term *TT* predictions, but also provides information on *TT* behaviour that is useful when making long-term predictions and for analysing its variability and identifying the factors that affect it.

In addition to this introduction, this paper contains four more sections. The section that follows is a review of short-term *TT* prediction models in the context of road-based mass transit systems. The proposed prediction model is described in the third Section. The results obtained through applying this model to a real case are presented in the fourth Section. The last Section presents the conclusions.

## 2. Related Works

This section reviews the proposals for short-term *TT* prediction models on routes run by public transport buses. Depending on the techniques used to carry out the predictions, these proposals may be classified into three groups: prediction models based on historical average models (*HAM*), prediction models based on machine learning regression (*MLR*), and prediction models based on state-based time-series (*STS*). Regardless of the theoretical foundations on which these prediction techniques are based, there are three common aspects. First, public transport routes for which *TT* predictions are to be made are modelled as a sequence of segments the endpoints of which may be stops or time control points. Second, the historical data used to implement the different proposals come from records obtained from the automatic vehicle location (*AVL*) systems and/or the automatic passenger counting (*APC*) systems installed in public transport vehicles. Third, short-term *TT* predictions for a public transport vehicle are done by taking as input the *TT* obtained by the vehicle on the route segments already travelled, and the data from the last vehicles to have travelled the route segments that said vehicle has not yet travelled. At this initial point of the review it should be mentioned that the works published on these prediction methods are generally lacking in their analysis of the input variables used to make *TT* predictions. We could, however, mention the works of Yetiskul and Senbil [3] and Comi et al. [4], which analysed the variables that affect *TT* behaviour in order to provide useful information for long-term *TT* planning. The former analysed *TT* behaviour in the Turkish city of Ankara. Using statistical variables, the authors studied how time, space and service factors affect *TT*. The latter was carried out in the city of Rome and consisted of an analysis based on time series designed to obtain *TT* behaviour patterns depending on time factors, and on how these patterns were influenced by traffic conditions. Based on this analysis, the authors developed a long-term *TT* prediction model based on time series. The review that follows is structured according to the theoretical model used by the proposed techniques, excluding those based on *HAM* because they were the initial proposals that have already been improved upon by proposals based on other more recent models.

The aim of the models based on *MLR* methods is to infer the value of a dependent variable, in this case *TT*, by means of a mathematical function based on a set of independent variables, this function being the result of a prior learning process. The techniques used in the studies include artificial neural networks (*ANN*); particularly multilayer perceptron (*MLP*) networks; support vector machine using the radial-basis function (*RBF*) as the kernel regression function; *k*-nearest neighbours (*KNN*) with the Euclidean distance being the most used metric; and decision tree regression (*DTR*). Yu et al. [5] proposed a prediction model based on *SVM*, using *RBF* as the kernel function. In this proposal, it is interesting to note that for the *TT* prediction not only is *TT* taken into account, but also an attribute that indicates the weather conditions (sunny day or rainy day) and the time of day in relation to demand (peak or off-peak). The *SVM* model uses an input vector consisting of three variables—the segment, the *TT* in the current segment, and the last *TT* value for the next segment—and the output data are the estimated times for the segments not yet travelled by the vehicle. Chang et al. [6] proposed a prediction model based on the *KNN* method. The data used are the records provided by the *AVL* systems. To make the prediction, the proposed model uses a set of historical data to obtain *TT* patterns, one for each day of the week; these patterns are called historical patterns. The vehicles that operate on the route for which *TT* is to be predicted communicate the times that they pass through the time control points to a central system. These are averaged, and a vector that characterises current *TT* for the route is obtained. To make the prediction, the historical *k* patterns closest to this vector are obtained using the Euclidean distance metric. Lee et al. [7] propose a *TT* prediction framework in which the vehicle’s arrival times, obtained from its *AVL* systems, are stored in a historical record. These historical records are processed using the *k*-means and *v*-means clustering techniques in order to obtain different groups that represent significantly different *TT* behaviours. In order to make the prediction, the times provided by the last vehicle that completed or is travelling on a route for which the *TT* are to be predicted are used as input data. Taking these current trajectory times to classify the *TT* to be predicted, the cluster with the pattern that most closely approximates the current times of the vehicle is sought. The *TT* predictions for the following route points will be the average *TT* from the data records that match this cluster. Gurmu et al. [8] proposes using an *ANN* model for real-time *TT* prediction using Global Positioning System (*GPS*) data. Gal et al. [9] proposes a combined method that uses a model based on queueing theory to obtain a first approximation of the prediction and a model based on *DTR*. The queueing theory model is based on the snapshot principle, using as input data the times that the last buses went through the stops. To implement the *DTR*-based method, the authors studied different techniques: random forest (*RF*), extremely randomised trees (*ET*), AdaBoost (*AB*), gradient tree boosting (*GB*) and an improved version of the gradient tree boosting technique (*GBLAD*), which produced the best results. Arhin and Stinson [10] proposes a prediction method based on a regression model that takes into account different factors (independent variables) that affect *TT* (dependent variable). These factors are passengers boarding, passengers alighting, passenger load, dwell time, segment length, bus stops, signalised intersections, access approaches and mid-segment crosswalks. The *TT* is the result of evaluating a function based on these factors, each weighted by a regression factor. Zhang et al. [11] uses a prediction method based on *SVM*. The data used in this proposal come from *AVL* systems. With historical records of traffic flow for different types of day and times of day, the authors constructed a training set to obtain a decision function that generates the prediction by combining current traffic data and the arrival times of the vehicles operating on the route.

Prediction methods that employ *STS* models are based on the assumption that the value of the variable to be predicted is a function of a linear or non-linear combination of the historical values of the state variable or variables. Shalaby and Farhan [12] proposes a prediction system based on Kalman filters (*KF*). The objective of this proposal was to predict *TT* between stops (nonstop running time) and the time that the vehicle spends at the stops on the route (dwell time). The data used are the records from the *AVL* and *APC* systems. Vanajakshi et al. [13] proposes a prediction method based on *KF*. The data used for the prediction are the *GPS* data obtained manually over 10 days because the buses did not have *AVL* systems. Song et al. [14] proposes a prediction model in which the *TT* between two consecutive stops on a route depends on the speed of the vehicle, which varies as it accelerates and decelerates, and by the time the vehicle is stationary because of traffic signals. This *TT* behaviour is predicted by an exponential smoothing function. In this proposal, predictions are made using two sets of data from two different sources: the records provided by the *AVL* systems and simulated Radio Frequency Identification (*RFID*) data. The public transport vehicles considered in this paper were buses and taxis. The authors concluded that with *RFID* data, better prediction results are obtained.

There are also proposals that use the *MLR* and *STS* models in combination. Chen et al. [15] proposes the combined use of an *ANN* model and a *KF* algorithm to predict *TT*. This model takes as input data the most recent information on the arrival time of the vehicle at a time point on the route and the prediction provided by the *ANN* model with historical data to predict, using a *KF* algorithm, the *TT* between two points on the route that have not yet been reached. The data were collected by *APC* systems. Bai et al. [16] proposes a combined prediction method in which an *SVM* model and a *KF* algorithm are used. The *KF* predictions were used to make short-term *TT* predictions. To do this, the prediction made by the *SVM* model and the *TT* taken by the last bus to travel along the segment of the route for which *TT* is to be predicted were taken as initial data. The authors compared the proposed model (*SVM-KF*) with four alternative models: an *ANN* model, a *KF* model, an *SVM* model, and a combined *ANN-KF* model. The results indicated that the proposed combined model, *SVM-KF*, performed better on the three road segments that were analysed.

Other studies have compared the different prediction models. In the specific context of a bus line in Macae (Brazil), Fan and Gurmu [17] conducted a comparative study on three of the most used models. To carry out this study, only data provided by the *AVL* systems were used. The models considered in this study were *HAM*, *KF* and *ANN*. Of the three models analysed, the *ANN* prediction model produced the best results. In the context of three Indian cities (Surat, Mysore, and Chennai), Jairam [18] analysed the behaviour of the *KNN*, *KF* and auto-regressive integrated moving average (*ARIMA*) prediction models. The study was conducted with the data provided by the *AVL* systems, analysing three routes (one in each of the cities). The models were analysed over a week and, from the results obtained, the authors concluded that the different prediction models provided similar results for route segments used exclusively by public transport vehicles. However, on segments of the route used by both public transport vehicles and private transport vehicles, the model that combined the *KNN-KF* techniques produced the best results. Hua et al. [19] compared prediction methods based on *ANN*, *SVM* and Linear Regression, introducing three Forgetting Factor Functions; the aim was to develop a prediction model using actual multi-route bus arrival time data from previous stops as inputs.

Table 1 shows the advantages and disadvantages of the different short-term *TT* prediction methods. Considering these properties, the method proposed in this article is characterised by providing a predictive power similar to *ANN*. In addition, the proposed method provides information on *TT* behaviour that can be used for planning schedules and is more easily applicable to all routes of a transport network than methods with greater predictive power.

## 3. Travel Time Prediction Model Based on Profile Similarity (*PSM*)

**Figure 1:**
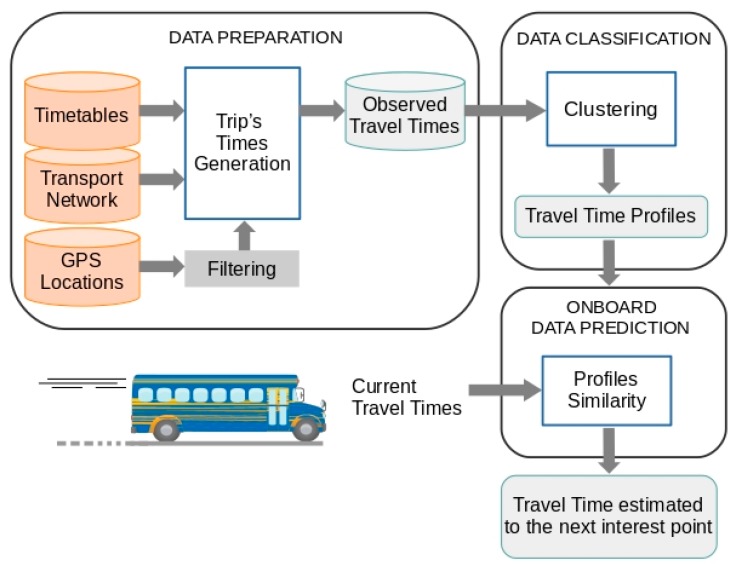
Overview of the *TT* prediction model.

As discussed in the previous section, in the context of regular road passenger transport, the short-term *TT* prediction methods take into account historical *TT* behaviour, modelled using different techniques such as *ANN*, *SVM*, *STS*, etc., and current behaviour, usually represented by the time taken by the vehicle to travel the last segment it has completed. The proposed method is based on the idea that it is possible to predict short-term *TT* using the representative elements from a classification process to represent historical behaviour, and the *TT* observed at all points of interest through which the vehicle has already passed to represent current *TT* behaviour (see Figure 1). Considering these working principles, the proposed prediction method has some interesting properties in relation to the techniques described in the review of the previous section. These properties are:

- Historical *TT* behaviour models that use patterns obtained through clustering techniques are much less costly, from the computational point of view, than the techniques usually used for this purpose, such as *ANN*, *SVM* and *STS*.
- The historical *TT* behaviour model can be applied to all routes on a transport network more easily than when using the alternative techniques mentioned above. In a *TT* prediction scenario for all the routes on a public transport network, representing historical *TT* behaviour by means of clustering techniques would first require the appropriate number of classes to be determined for each route. This could be done systematically using metrics that measure the quality of the resulting clusters. However, the use, for example, of *ANN* or *SVM*, to model *TT* behaviour on all the routes would require different configurations that would have to be obtained through learning and validation processes.
- A third advantage, resulting from the two previous advantages, is that the continuous evolution of the representation of historical behaviour by means of clustering techniques is less costly than with the alternative techniques mentioned above. This continuous evolution is necessary because *TT* depends on external factors that are variable over time.

For the representative elements obtained through a clustering process to reflect historical *TT* behaviour, a significant sample of *TT* is needed. As explained in the review of the previous section, there are mainly two data sources from which to obtain a sample of historical *TT* values: *AVL* and *APC*. Although the proposed method is independent of the data source that is used to obtain the historical *TT* data records, in the use case presented in the fourth Section *AVL* systems are used as the data source. In this implementation, the basic data are the *GPS* locations of the vehicle. In order to carry out this analysis of the routes, it is also necessary to handle data of a different nature, which are typically used in public transport operations (transport network design and operations control).

### 3.1. Formal Model Framework

The objective of the proposed method is to estimate short-term *TT* in a context of regular passenger road transport planned by timetable. As already mentioned above, this type of *TT* prediction is executed and is valid during the trip made by a public transport bus as it operates a particular route. In this section, entities related to the proposed method are presented and formalised. Table 2 includes the notation used for the model entities.

#### 3.1.1. Definition of the Entities Used by the Model

**Figure 2:**
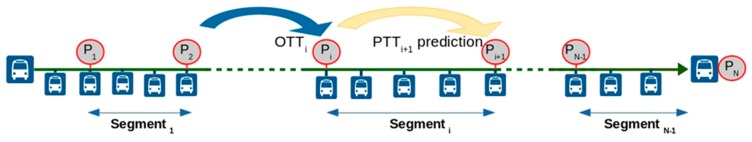
Schematic representation of a route according to the model.

The first entity to be formalised is the public transport line. For the purposes of this paper, a line is defined as systematic, scheduled route taken by public transport buses. Systematic means that the bus always follows the same route and stops at a series of pre-established stops that do not vary. Scheduled means that there is a schedule that establishes when the buses must run the route. The operation of a line by a public transport vehicle shall be termed a trip. In the model, *L* represents a generic line and a specific line is specified by means of the notation *L_c_*, where the subscript *c* is an integer value that uniquely identifies the line. Trips on *L_c_* are specified by means of the notation *E_c_*, where *c* is the identifier of the line. In the model, time is specified by the notations *T* and *t*. *T* represents a time interval and *t* represents a moment of time, which is the minimum unit of time. All trips by line *L_c_* that have been made over a period of time *T* are specified by the notation *E_c,T_*. Similarly, a trip that has begun at a moment of time *t* is specified by means of the notation *e_c,t_*. Stops on the route of *L_c_* are represented by the notation *S_c_*. In the context of this study, stops on a route for which the *TT* is to be predicted are called points of interest and are represented by *P_c_*. Points of interest on the route of *L_c_* are designated by the notation *P_c,i_*, where the subscript *i* identifies the point of interest and its value matches the order in which the bus passes them following the planned route. For example, *P*_*c*,1_ is the first point of interest through which the vehicles pass when operating route *c*. When selecting points of interest, the only restriction is that the first stop of the route cannot be a point of interest. The section of the route that runs from one point of interest to the next is called a route segment (see Figure 2). In this figure, the blue rectangles represent the stops and the red circles, the points of interest.

In the context of regular passenger road transport, *TT* is the result of the sum of two times: dwell time, *DT*, and nonstop running time, *RT*. *DT* represents the time that the vehicle is stationary at stops for passengers to board or alight from the vehicle. *RT* represents the time taken by the vehicle to go from one stop on the route to the next. If a route has *N* stops, then the total *TT* of a trip is:

(1) $$TT=\overset{N}{\underset{n=1}{\sum }}DT_{n}+\overset{N-1}{\underset{n=1}{\sum }}RT_{n}$$

The term arrival time is the time at which the vehicle arrives at that stop. The arrival times observed at the points of interest on trip *e_c,t_* are represented by *OPT_c,t_*. If *L_c_* has *N* points of interest, then the arrival times of each entity *OPT_c,t_* are recorded as an array of *N* integer values. The set comprising the arrival times on all trips on a route completed by *L_c_* in a time period *T* is represented by *OPT_c,T_*. The prediction made by the proposed model consists of estimating the time that elapses between the vehicle reaching point of interest *i* and point of interest *i* + 1. Therefore, the *DT* at point of interest *i* is included in this time. 

#### 3.1.2. The *k*-Medoid Clustering Technique

Clustering methods are classification techniques that group large sets of elements, characterised by a set of attributes, using similarity criteria. For the purposes of this study, clustering techniques have two interesting properties. The first is that they do not require prior learning and the second is that there are metrics that measure the quality of the resulting clusters. In the proposed model, assuming the formulation stated above, given a significant period of time *T* and a route *L_c_*, the set of elements to be classified is composed by all the trips on route *L_c_* that have been completed in period *T*—i.e., dataset *E_c,T_* representing all trips *e_c,t_*. These trips are represented by the *TT* observed at each point of interest *OPT_c,t_* and the dataset of all the representations of all trips *E_c,T_* is represented by *OPT_c,T_*. If in the prediction of *TT*, *n* points of interest have been defined on *L_c_*, then each element *OPT_c,t_*, is represented by an *n*-tuple (*TT*_1_, *TT*_2_, …, *TT_n_*), which corresponds to the *TT* observed on trip *e_c,t_*.

In the proposed methodology, the historical *TT* behaviour profiles are obtained by the *k*-medoids method [20]. This method belongs to the group of non-hierarchical clustering techniques, and any distance metric can be used to measure the similarity between two elements. The grouping criterion is to cluster data around the most representative objects, called the “medoid”, of the dataset. The most representative object is the most centrally located point. Therefore, this representative object is a pre-existing object from the sample and not an object that is generated in the classification process. This property means that the *k*-medoids technique responds well in the event of outliers. The most used distance metrics in the *k*-medoids technique are the Euclidean distance and Manhattan distance metrics. Equations (2) and (3) express the Euclidean and Manhattan distances, between two objects, *X_i_* and *X_j_*, for a set of *Q* objects, each object being represented by *n* attributes. Equation (2) is the Euclidean distance and Equation (3) the Manhattan distance.

(2) $$d_{ij}=\sqrt{\overset{n}{\underset{p=1}{\sum }}{\left(X_{ip}-X_{jp})\right)}^{2}}\;\;i=1,\;\ldots ,\;Q;\;j=1,\;\ldots ,\;Q$$

(3) $$d_{ij}=\overset{n}{\underset{p=1}{\sum }}\left|X_{ip}-X_{jp}\right|\;i=1,\;\ldots ,\;Q;\;j=1,\;\ldots ,\;Q$$

**Figure 3:**
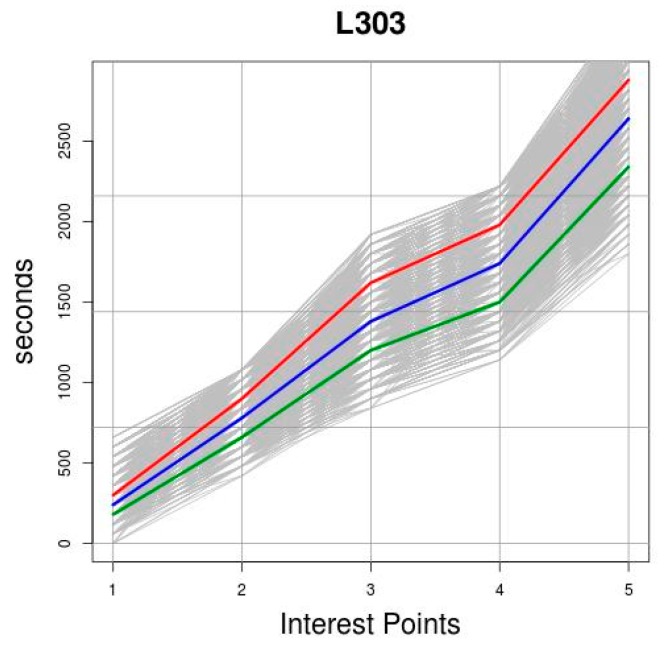
Representation of trip data and resulting medoids.

An example of clustering using the *k*-medoids technique is shown in Figure 3. On the horizontal axis the points of interest are represented and on the vertical axis the observed *TT*, measured in seconds, at the points of interest of a route. Each grey curve represents the *TT* recorded for a trip, *OPT_c,t_*. The dataset *OPT_c,T_* is formed by all the *OPT_c,t_* curves. The red, blue and green curves represent the medoids, which are the representative object of each of the three resulting clusters.

To evaluate the validity of a clustering solution there are different criteria, which may be classified into three categories: external indices, which measure the extent to which cluster labels match externally-supplied class labels; internal indices, which measure the intrinsic information of each dataset; and relative indices, which are used to compare several different clustering solutions. For the purposes of this study, an internal index was chosen to measure the quality of the clusters: the silhouette function [21]. This measures the consistency of the cluster based on a comparison of the tightness and separation of the elements of each segment generated. This is computed by the following expression:

(4) $$S_{i}=\{\begin{array}{ll} 1-\frac{A_{i}}{B_{i}}, & if\;A_{i}<B_{i}\\ 0, & if\;A_{i}=B_{i}\\ \frac{B_{i}}{A_{i}}-1, & if\;B_{i}<A_{i} \end{array}$$

In expression (4), *A_i_* is the average distance from object *i* to the other objects within the cluster and *B_i_* is the smallest average distance from *i* to all the objects of each of the clusters to which *i* does not belong. The values returned by the silhouette function are in the range −1 and 1. A value close to 1 indicates a high degree of consistency in the resulting clusters and, conversely, a value close to −1 indicates that the resulting clusters have little consistency.

### 3.2. Travel Time Prediction Scheme

Taking the concept of point of interest, if we assume that a vehicle is at point *P_i_* of the sequence of points of interest on route *L_c_*, the objective of the proposed method is to estimate the *TT* required to reach the next point in the sequence of points of interest of the route, i.e., the *TT* to reach point *P_i+_*_1_. The recent history for that vehicle, located at point of interest *P_i_*, is represented by a set of ordered values that represent the *TT* observed when going through the points of interest already travelled, represented by the *i*-tuple (*TT*_1_, *TT*_2_, …, *TT_i_*). Past history, in period *T*, is represented by the medoids resulting from applying the *k*-medoid clustering technique to the set *OPT_c,T_*, these medoids represent the historical profiles of the *TT*. When the vehicle is at *P_i_*, the *TT* to reach point *P_i+_*_1_ is estimated in two steps. In the first step, from the observed *TT* (*TT*_1_, *TT*_2_, …, *TT_i_*) the medoid that has the most similar behaviour to the *TT* behaviour recorded up to point *P_i_* is selected. The similarity metric used is the same as that used in the clustering process through which the medoids were obtained. Once the most similar medoid has been selected, then the prediction of the *TT* for segment *S_i_*, i.e., to reach point *P_i+_*_1_, is:

(5) $$PTT_{i+1}=TT_{i}+M_{k,i+1}-M_{k,i}$$

In Equation (5), *PTT_i+_*_1_ represents the prediction of the *TT* taken to reach point *P_i+_*_1_. *TT_i_* is the *TT* observed at point *P_i_* and *M_k_* is the medoid to which the recorded *TT* are most similar. *M_k,i_* and *M_k,i+_*_1_ represent the attributes *i* and *i* + 1 of this medoid, i.e., the *TT* that represent the historical behaviours at points *P_i_* and *P_j+_*_1_ of the *k* cluster.

Next, the short-term *TT* prediction algorithm is described for trip *e_c,t_* that is completed in vehicle V.

Data:

- *K*: number of clusters used to represent historical *TT* behaviour;
- {*M*_1_, …, *M_k_*}: set of *k*-medoids that represent *TT* behaviour on trips on route *L_c_*;
- *i*: last control point through which V has passed on trip *e_c,t_*;
- *TT*_1→*i*_: observed arrival time at interest points 1, 2, …, *i* on trip *e_c,t_* (recent behaviour);
- *M*_*k*,1→*i*_: for the *k*-medoid, values of the arrival time at interest points 1, 2, …, *i* (historical behaviour);
- *PTT_i+_*_1_: objective of the algorithm, which is to predict *TT* at interest point *i* + 1.

Step 1. Obtain the medoid that most resembles the observed *TT* behaviour up to the last interest point that V has passed on trip *e_c,t_*. Dist(a,b) is the function that evaluates the distance metric that is used to assess similarity.

*D_min_* = ∞

For (*j* = 1 to *K*) do

Do

  *D* = Dist(*M*_*k*,1→*i*_, *TT*_*i*→1_)

  If (*D* < *D_min_*)

  Begin

    *k* = j

    *D_min_* = *D*

  End

End

Step 2. Estimate *PTT_i+_*_1_

*PTT*_*i*+1_ = *TT_i_* + *M*_*k*,*i*+1_ − *M*_*k*,*i*_

**Figure 4:**
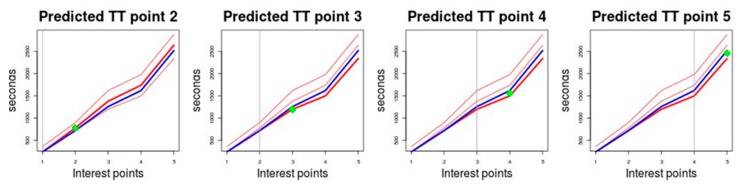
Schematic representation of the prediction method.

The prediction method is illustrated graphically in Figure 4. This illustrates the prediction for a route on which five points of interest have been defined and that are represented on the horizontal axis. The *TT* measured in seconds is represented on the vertical axis. The historical behaviour is represented by three medoids that have been drawn in red. The blue graph represents the *TT* of the test trip for which the prediction is being made and the green dot is the *TT* value predicted for the different points of interest. Table 3 illustrates the prediction method numerically with an example prediction of a trip. The route in this example has five points of interest, columns *P*_1_, *P*_2_, *P*_3_, *P*_4_ and *P*_5_. The historical *TT* behaviour is represented by three medoids, rows *M*_1_, *M*_2_ and *M*_3_. The observed *TT* up to each of the points of interest is displayed in row *TT*. As the vehicle reaches each point of interest, the medoid with the behaviour that is most similar to the *TT* observed on the trip is selected, using Manhattan distance as the similarity metric. Rows *D*(*TT*,*M*_1_), *D*(*TT*,*M*_2_) and *D*(*TT*,*M*_3_) contain the values resulting from calculating this distance between *TT* and *M*_1_, *M*_2_ y *M*_3_, respectively. At point of interest *P*_1_, we calculated the distance between the *TT* observed at this point, which was 180, and the values of the medoids at this point, which were 360 for *M*_1_, 240 for *M*_2_ and 240 for *M*_3_. The Manhattan distance values obtained were 180 for *D*(*TT*,*M*_1_), 60 for *D*(*TT*,*M*_2_) and 60 for *D*(*TT*,*M*_3_). Since the minimum distance value obtained was 60 and it was obtained with two medoids, *M*_2_ and *M*_3_, the first medoid that produced this minimum value, in this case *M*_2_, was selected for this method. The predicted *TT* values at each point *P_i_* are displayed in row *PTT*. The *TT* prediction to reach point of interest *P*_2_ will be made taking medoid *M*_2_ as the historical reference and the prediction will be calculated, according to Equation (5), by adding to the *TT* observed at *P*_1_, which is 180 s, the difference between the *TT* value of *M*_2_ at point *P*_2_, which is 780 s, and the value of *M*_2_ at point P_1_, which is 240 s. Therefore, the value of the *TT* prediction to reach P_2_ is 720 s. The *TT* prediction to reach point *P*_3_ would be carried out analogously to this, but in this case the *TT* profile on the trip will consist of two values (180,720) and the historical *TT* behaviour profiles will be (360,900) according to *M*_1_, (240,780) according to *M*_2_ and (240,720) according to *M*_3_. At this point the historical profile that is most similar to the profile observed on the trip is that represented by medoid *M*_3_. As shown in Table 3, at the rest of the points of interest, *M*_3_ is the medoid with a profile most similar to the observed *TT* values and therefore its *TT* values will be those used to make the predictions at the other points.

## 4. Results and Discussion

The main objectives of the tests were to select the most suitable parameters for the proposed method, to evaluate its prediction accuracy and to compare it with other methods. To make this analysis more complete, data were collected from two bus lines with different characteristics in terms of demand, route length, types of road and the population centres that they run through. 

In terms of resources and tools, a computer with an Intel(R) Core (TM) i7-2600K CPU processor @ 3.40GHz with 16 GB RAM was used. Oracle DB—the database environment used by the transport operator that provided its data—was used to prepare the data. For the modelling phase, the RStudio framework was used, specifically Cluster Package [22] and Neuralnet Package [23]. To visually map the data, the GoogleMap framework was used. 

### 4.1. Description of Datasets

**Figure 5:**
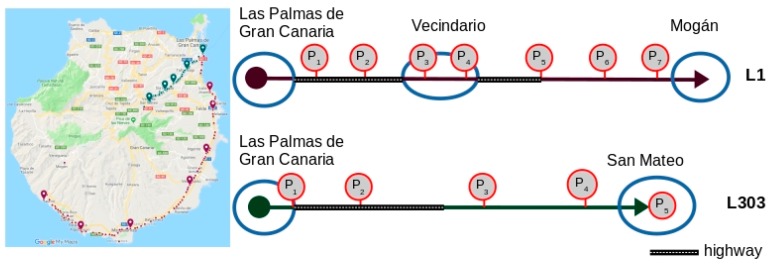
Representation of the two lines analysed.

The datasets used came from the operational records of two lines operated by the intercity public transport network of the Island of Gran Canaria. The period of time analysed in the study was the whole of 2015. Therefore, according to the model, the value of *T* is the year 2015. These two lines, identified by codes *L*_1_ and *L*_303_, cover two important transport corridors on the Island. Line *L*_1_ covers the Capital-South corridor, starting in the city of Las Palmas de Gran Canaria, which, as the most populated urban nucleus where the main public service centres of the island are based, is the main transport generation point. The line goes through the main tourist centres—tourism is the largest sector of the island economy and generates the most employment. Line *L*_303_ covers the Capital-Centre corridor, starting in the city of Las Palmas de Gran Canaria and travelling through the largest population centres in the centre of the island. Table 4 shows the length and number of stops on these routes, and the number of stops selected for the study—the points of interest. In the case of line *L*_1_, of the 91 stops, 7 points of interest were selected. In the case of line *L*_303_, six were selected, giving rise to five route segments. Two aspects were taken into account when selecting stops as points of interest: the number of travellers and the distance between the stops (stops very close to each other were ruled out). The routes taken by the two lines travel along different types of road (fast roads, rural roads and urban roads) and, due to the different motivations and type of user, moments of peak and off-peak demand vary. Figure 5 shows the routes of these lines. On the left side of the figure, the route is shown on a map of the island, where the dots marked on each route represent the stops, and the balloon markers the points of interest selected for the study. On the right side, a schematic representation of the routes is displayed.

The basic data unit used was the *GPS* location data that each bus automatically records every minute. In order to obtain all the *TT* for the trips completed in 2015, a data mining process was carried out to reconstruct all the trips on the routes to be analysed. Table 5 shows the number of location records used to reconstruct the route trips (*NGPS*), the number of reconstructed trips (*NRE*) and the number of these trips for which the routes are consistent with the planned route and do not have errors (*NCRE*). The set of correct trips is dataset *E*_1,2015_ in the case of *L*_1_, and *E*_303,2015_ for *L*_303_.

### 4.2. Performance of the Method Using Different Configuration Parameters

**Figure 6:**
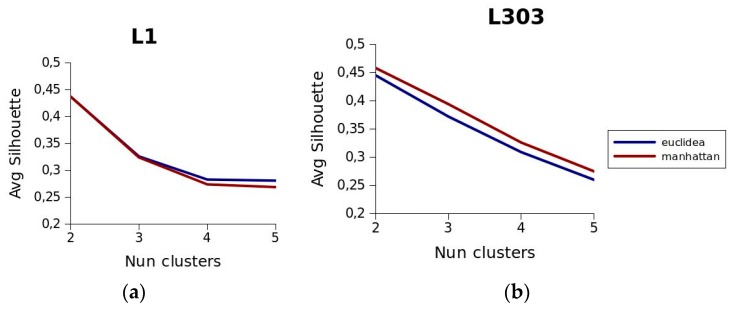
Behaviour of the silhouette function for each route depending on the number of clusters used in the clustering process. (**a**) Route *L*_1_; (**b**) Route *L*_303_.

**Figure 7:**
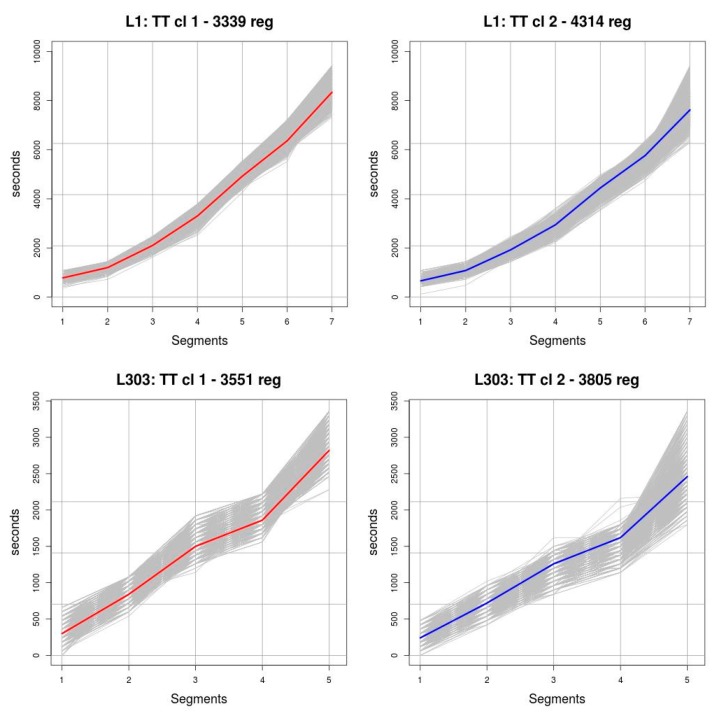
Clusters resulting from applying the *k*-medoids technique with two clusters to the *TT* observed on the trips.

The first phase of the tests was designed to check how the distance metric and the number of clusters affected the process of creating the clusters. Two distance metrics were tested: the Euclidean and the Manhattan. These metrics were used to generate different segments in the *OPT*_1,2015_ and *OPT*_303,2015_ datasets, specifying different numbers of clusters beforehand, and evaluating the quality of the resulting segments with the silhouette function. Figure 6 shows the values returned by the silhouette function. The number of clusters used is represented on the horizontal axis and the resulting value of the function on the vertical axis. Based on the results obtained, we decided to use the Manhattan distance as the distance metric and to partition the data into two clusters. As can be seen, the behaviour of this function is very similar in the case of route *L*_1_. However, it may be observed in the case of route *L*_303_ that the function produces higher values, that is, higher levels of consistency in the clusters, when the Manhattan distance is used. Regarding the number of clusters, the best levels of consistency, regardless of the route analysed and the metric used, occur when two clusters are used. Indeed, both for route *L*_1_ and for route *L*_303_, the values of the silhouette function are very close to 0.45 using both the Euclidean metric and the Manhattan metric. It may also be observed that, irrespective of the route analysed and the distance metric used, as we increase the number of clusters used in the clustering process, the value of the silhouette function decreases, which means that the degree of consistency decreases. Figure 7 shows the result of grouping routes *L*_1_ and *L*_303_ using two clusters. The objects of the clusters are drawn in grey and the medoids of each cluster are drawn in red and blue. These medoids are the ones used in *TT* predictions that are displayed as results.

### 4.3. Evaluation of Prediction Accuracy

The metric used to evaluate prediction accuracy for a trip was the mean absolute percentage error (*MAPE*), which is expressed according to Equation (6). In this equation, *N* represents the number of points of interest on the route, *i* varies between 1 and the total number of segments. *OPT_i_* is the *TT* recorded for segment *i* and *PTT_i_* is the predicted *TT* for segment *i*. Both O*PT_i_* and *PTT_i_* are expressed in seconds. For this evaluation, all *OPT*_1,2015_ and *OPT*_303,2015_ data records were used for the samples.

(6) $$MAPE=\frac{1}{N-1}\;\overset{N-1}{\underset{i=1}{\sum }}\frac{\left|PTT_{i}-OPT_{i}\right|}{OPT_{i}}$$

Table 6 shows the results obtained for the trips completed by each bus line. The *AV_MAPE_* row represents the average value for all the trips, and the S_i_ rows show the average *MAPE* value for each segment of the route. With regard to *L*_1_, the worst prediction was made for segment *S*_1_, since this is where the highest *MAPE* value (0.1549) was obtained: on this segment the percentage of error in *TT* prediction in relation to the observed value was 15.49%. The best prediction was made for segment *S*_4_, where an error percentage of 7.61% was obtained. The average value, considering the *MAPE* values for all segments of the route—*AV_MAPE_* row—was 0.1106. Therefore, considering the *MAPE* values for all the segments, *PSM* commits an error of, on average, 11.06% of the observed *TT* values. With regard to *L*_303_, *S*_2_ was the segment for which the highest *MAPE* value (0.223) was obtained: on this segment the percentage of error in the prediction, in relation to the observed value, was 22.49%. The best prediction was made for *S*_4_, where the lowest *MAPE* value (0.0939) was obtained: an error percentage of 9.39% in relation to the observed *TT* values on that segment. The average value, considering the *MAPE* values for all segments of the route—*AV_MAPE_* row—was 0.1325, i.e., the average error rate was 13.25% in relation to the *TT* observed for all segments. 

### 4.4. Comparison with Other Prediction Methods

*PSM* was compared with two reference methods that are widely used in the proposals described in the section on short-term *TT* prediction methods. These alternative methods are: predictions based on the average TT obtained from the observed *TT* (*AvgTT*) and an *ANN* prediction model. The analysis consisted of comparing the *MAPE* values obtained from these alternative methods with the values provided by the proposed method. The resulting *MAPE* values were compared on each segment of the analysed routes as well as the global value obtained for all the routes. The required processing times of each method were also compared.

The *ANN* model used for the comparison was the *MLP* network, analogous to that used in [6]. The input variables for the network were time of day, the identification of the point of interest *i* that the vehicle has arrived at, and the *TT* observed since the beginning of the route. The output variable of the network was the *TT* of the vehicle in segment *i*. The dataset used for training the two *ANN*s, one for each route, was built with 80% of the data records from the *OPT*_1,2015_ and *OPT*_303,2015_ datasets and the test dataset used the remaining 20%. Based on the results of the tests, using three, four and five nodes in the hidden layer, the configuration with three nodes was chosen as the configuration with the best results.

#### 4.4.1. Comparison of Prediction Accuracy 

**Figure 8:**
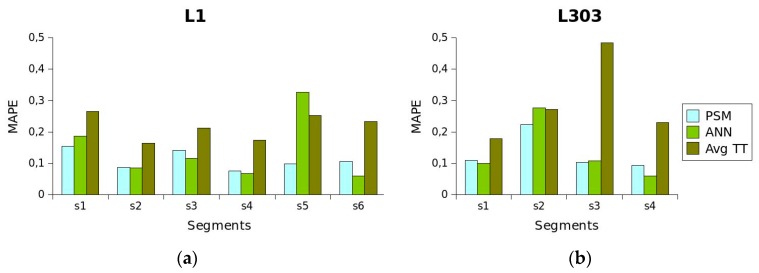
MAPE values obtained by applying the three methods. (**a**) Values obtained for *L*_1_. (**b**) Values obtained for *L*_303_.

Figure 8 shows the results obtained by applying each of these methods to the two routes analysed. As shown in Figure 8a, the results obtained for route *L*_1_ clearly indicate that *AvgTT* produces the worst results. *PSM* provided better results for *S*_1_ and *S*_5_ compared to the *ANN* and *AvgTT* methods, obtaining a considerably better prediction for S_5_ segment. *ANN* produced better results for *S*_3_ and *S*_6_. On *S*_2_, the *MAPE* values obtained by the *PSM* and *ANN* methods were practically the same. If the *MAPE* values obtained for all the segments are taken into account, the conclusion drawn is that *PSM* produces values that vary less than those produced by *AvgTT* and *ANN*. *PSM* performed the worst on *S*_1_, with an *MAPE* value close to 0.15. The best *PSM* performance was obtained on *S*_4_, with a value close to 0.07. In the case of *ANN*, it performed best on *S*_6_, with a value close to 0.06, and worst on *S_5_*, with a value close to 0.32, which even exceeded the value obtained for that segment by *AvgTT*. The results obtained for route L_303_ are shown in Figure 8b. These results also clearly indicate that *AvgTT* provides the worst results. *PSM* produced better results than *AvgTT* and *ANN* on segments *S*_2_ and *S*_3_. *ANN* produced better results on segments *S*_1_ and *S*_4_. As was the case with line *L*_1_, if the *MAPE* values obtained for all the sections are taken into account, *PSM* produces values that vary less than those produced by *AvgTT* and *ANN*. It can be seen that the worst performance of *PSM* was on segment *S*_2_, with a *MAPE* value close to 0.22. For the rest of the segments, the values obtained were similar and close to 0.10. In the case of *ANN*, the best performance was on segment *S*_4_, with a value close to 0.05, and the worst on *S*_2_, with a value close to 0.27, very close to the value obtained for that segment by *AvgTT*.

**Figure 9:**
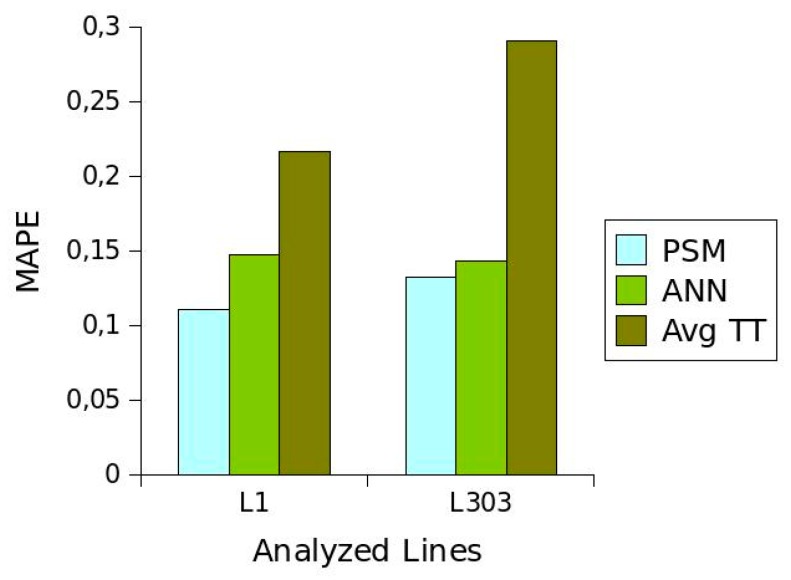
Average *MAPE* values for each method on each of the lines studied.

Figure 9 shows the average *MAPE* values obtained for each segment of each of the routes analysed. It can be seen that *PSM* performed the best since it provided a value close to 0.11 on *L*_1_ and 0.13 on *L*_303_. In the case of *ANN*, the average *MAPE* values obtained for the two routes were similar, and close to 0.14. The worst-performing method was clearly *AvgTT* with values of 0.21 and 0.29 for *L*_1_ and *L*_303_, respectively.

#### 4.4.2. Comparison of Complexity of the Required Processes and Computation Time 

Although substantial differences were not observed between the results obtained by *PSM* and *ANN* in all cases, certain aspects that make *PSM* more attractive need mentioning. First, it is not necessary to normalise the data, as is the case of *ANN* where this needs to be done to. On the one hand, avoid differences between the magnitudes of the variables that affect the calculation of the weightings during the training phase. On the other hand, adapt them to the corresponding ranges of the different activation functions. Second, there are considerable differences between the time used to create the *TT* profiles used by *PSM*, and the time spent on the *ANN* training process. For example, on one of the analysed routes the training of the network took 59 times longer than the clustering process, as can be seen in Table 7, where the times spent on each case are shown. Third, while it is possible to find evaluation functions for the generated clusters, the number of nodes of the hidden layer has to be determined in the experimental phase, so it is easier to find an optimal configuration in the *PSM* model. Fourth and lastly, *ANN* errors have greater variability, as can be seen especially in the case of line *L*_1_ (Figure 8a).

### 4.5. Final Discussion 

Considering the results, it can be said that *PSM* provides a prediction accuracy similar to that provided by *ANN*. However, *PSM* is more stable behaviour in the accuracy of its predictions. In addition, the behaviour patterns represented by the medoids used in *PSM* provide interpretable information about *TT*, an advantage that *ANN* and other models with high predictive power—described in Table 1, such as proposals based on *SVM*—do not have. Considering another property shown in Table 1—the ease of applying the method to all the routes on a transport network—*PSM* is easier to apply, with lower computational cost, than models based on *ANN*. In this regard, the computational cost of applying *PSM* to *TT* predictions on all routes of a transport network is considerably lower than that of applying techniques based on *ANN*. This is because *PSM* does not require training processes for its application, whereas *ANN*-based prediction systems do require learning, which would also have to be done for each route of the transport network. Given the characteristics of the *SVM*-based methods—another method with high predictive power—*PSM* would also be easier to apply to all the routes of a transport network, with a lower computational cost.

As with the *ANN*-based methods, the predictions made by *PSM* in situations involving exceptional conditions, such as for example abnormally slow speed of the vehicle due to traffic incidents, have a lower accuracy. For this reason, the combined prediction methods *ANN-KFM* or *SVM-KF* were used. Therefore, it may be inferred that the use of a combined *PSM-KF* model could be used in contexts in which recent historical information provided by other vehicles was available, as in the case of urban transport. Finally, we would like to underscore the fact that the low computational cost of making *TT* predictions with *PSM* would enable predictions to be calculated on the vehicle itself.

## 5. Conclusions

In this article, a short-term *TT* prediction model for public transport buses is proposed. To estimate *TT*, the model takes into account the historical behaviour of *TT*, represented by the medoids resulting from a clustering process based on the *k*-medoids technique, and the current *TT* behaviour, which is represented by the *TT* observed on the vehicle for which the prediction is made. Therefore, the method does not require recent *TT* data provided by other vehicles, meaning that it can be executed autonomously on the vehicles themselves. The method has been applied in two real cases of public transport lines of different characteristics. The results show that, in general, the average error made in the predictions is around 13% of the observed *TT* values, a result similar to that obtained by the alternative method *ANN*, although its variability is less than this alternative method. The proposed method has some interesting features. The first is that, since it does not need recent *TT* data provided by other vehicles, the method can be used in the context of line services planned by timetable, which is the planning method used for intercity transport. Second, the method not only provides short-term *TT* estimates; by using the *k*-medoids clustering technique to extract *TT* patterns from bus trips, it also provides information on its behaviour, thus helping to make long-term *TT* predictions, which are used in the planning of line services. Both from the computational point of view and the configuration of the required parameters, the method is less costly than the most widely used alternative methods, based on machine learning regression or state-based time-series, because it does not require costly learning processes and validation. Therefore, the prediction of short-term *TT* on all the routes of a transport network can be carried out by the proposed method at considerably lower cost.

## Figures and Tables

**Table 1:** Advantages and disadvantages of short-term *TT* prediction models.

Model	Technique	Advantages	Disadvantages
***MLR***	*ANN*	Predictive power Ability to discover non-linear relationships	Low interpretability High volumes of data are required Poor scalability for handling large volumes of data High cost for applying to all the lines of a transport network
	*SVM*	Predictive power Ability to discover non-linear relationships	Low interpretability High volumes of data are required Poor scalability for handling large volumes of data High cost for applying to all the lines of a transport network
	*KNN*	Non-parametric Handling missing data and outliers	Low interpretability High volumes of data are required
	*DTR*	Non-parametric High scalability for handling large volumes of data Interpretability	Poor predictive power
***STS***	*KF*	Ability to filter noisy data Ability to react to unexpected events	Based on the most recent data samples
	*ARIMA*	High computational speed	Highly sensitive to outliers
	Smoothing functions	Simplicity	Poor predictive power
Flow Conservation and Traffic Dynamic	Queueing theory	Realistic models for complex realities	Independent of the input data distribution

**Table 2:** Notation used for the model entities.

Travel Time	*TT*
Generic line of public transport	*L*
Specific line of public transport identified by code c	*L_c_*
Set of trips of *L_c_*	*E_c_*
Set of trips of *L_c_* made during the period *T*	*E_c,T_*
Orderly set of bus stops of *L_c_*	*S_c_*
Orderly set of interest points of *L_c_*	*P_c_*
*i*-th point of interest of *L_c_*	*P_c,i_*
Trip of *L_c_* that starts at the instant *t*	*e_c,t_*
Arrival times observed at the points of interest on trip *e_c,t_*	*OPT_c,t_*
Set comprising the arrival times on all trips of *E_c,T_*	*OPT_c,T_*
Dwell time	*DT*
Dwell time at bus stop *n*	*DT_n_*
Nonstop running time	*RT*
Nonstop running time in segment n of the route	*RT_n_*
Observed arrival time at *i*-th point of interest on the current trip	*TT_i_*
Predicted arrival time at *i*-th point of interest on the current trip	*PTT_i_*

**Table 3:** Numerical illustration of a *TT* prediction on a route with four points of interest (*P_i_*) and using three medoids (*M_i_*). *TT* row is the observed *TT*s and *D*(*TT,M_i_*) is the Manhattan distance.

	*P*_1_	*P*_2_	*P*_3_	*P*_4_	*P*_5_
***M*_1_**	(360)	(360,900)	(360,900,1620)	(360,900,1620,1980)	(360,900,1620,1980,2880)
***M*_2_**	(240)	(240,780)	(240,780,1380)	(240, 780, 1380,1740)	(240,780,1380,1740,2640)
***M*_3_**	(240)	(240,720)	(240,720,1200)	(240,720,1200,1500)	(240,720,1200,1500,2340)
***TT***	(180)	(180,720)	(180,720,1260)	(180,720,1260,1620)	(180,720,1260,1620,2460)
***D*(*TT*,*M*_1_)**	180	360	720	1080	1500
***D*(*TT*,*M*_2_)**	**60**	120	240	360	540
***D*(*TT*,*M*_3_)**	60	**60**	**120**	**240**	**360**
***PTT***		720	1200	1560	2460

**Table 4:** Length and number of stops on the lines considered in the use case.

	*L*_1_	*L*_303_
Length (km)	60	32
Number of stops	91	42
Points of Interest	7	5

**Table 5:** Number of records from each of the datasets for the preparation phase.

	*L*_1_	*L*_303_
***NGPS***	2,038,668	615,813
***NRE***	11,847	9887
***NCRE***	8419	7862

**Table 6:** MAPE values for each of the segments of the analysed routes.

	*L*_1_	*L*_303_
***AV_MAPE_***	0.1106	0.1325
***S*_1_**	0.1549	0.1098
***S*_2_**	0.0868	0.2232
***S*_3_**	0.1413	0.1029
***S*_4_**	0.0761	0.0939
***S_5_***	0.0978	
***S*_6_**	0.1067	

**Table 7:** Time required by the methods, in seconds.

	*L*_1_	*L*_303_
***PSM***	6572	9046
***ANN***	385.39	74,824
